# Improvement of Cutaneous Wound Healing via Topical Application of Heat-Killed *Lactococcus chungangensis* CAU 1447 on Diabetic Mice

**DOI:** 10.3390/nu13082666

**Published:** 2021-07-31

**Authors:** Yohan Nam, Jonghwa Kim, Jihye Baek, Wonyong Kim

**Affiliations:** Department of Microbiology, College of Medicine, Chung-Ang University, Seoul 06974, Korea; skadygks12@naver.com (Y.N.); ksjhjs@hanmail.net (J.K.); jihye2258@naver.com (J.B.)

**Keywords:** *Lactococcus chungangensis* CAU 1447, postbiotics, heat-killed probiotics, wound healing, type 1 diabetes mellitus

## Abstract

Cutaneous wound healing comprises a complex systemic network. Probiotics, naturally extracted substances, medicine, and chemical compounds have been used for wound healing, but the application of postbiotics as therapeutic agents has yet to be explored. Our study shows potential beneficial effects of heat-killed *Lactococcus chungangensis* CAU 1447 on type 1 diabetic mice. The postbiotic strain significantly decreased the skin wound size. The activity of myeloperoxidase secreted from neutrophils also decreased. The molecular mechanism of wound healing was adjusted by important mediators, growth factors, chemokines, and cytokines. These elements regulated the anti-inflammatory activity and accelerated wound healing. To determine the role of the postbiotic in wound repair, we showed a similar taxonomic pattern as compared to the diabetic mice using skin microbiome analysis. These findings demonstrated that heat-killed *Lactococcus chungangensis* CAU 1447 had beneficial effects on wound healing and can be utilized as postbiotic therapeutic agents.

## 1. Introduction

The fundamental blockade of the skin’s immune response plays an important role in human health [1]. The skin is a physiologically important organ, as it modulates homeostasis and balance [2]. The loss of skin function, which ensures the integrity of a large proportion of the body, causes wounds and illness and initiates the healing process [1].

Cutaneous wound healing is a general and complicated process that interacts with biomolecular and cellular mechanisms to recover impaired wound tissue to its native state [3]. The principal biological wound healing processes include matrix deposition, leukocyte recruitment, epithelialization, regulation of inflammatory responses, and vigorous angiogenic reactions [4]. Among them, angiogenesis plays an important role in the wound healing process and is involved in the growth factor regulation, pro-inflammatory cytokine secretion, and gathering of repair cells [5,6]. Additionally, interactions involving skin flora during wound healing are considered useful because they facilitate innate immune responses [7]. This complex wound healing process occurs at an adequate rate in healthy people; however, people with diabetes show delayed angiogenesis and a different healing process because of their reduced internal body activity compared to healthy people [8,9].

Research on wound healing has used many materials, such as probiotics, various natural substances, medicines, extracted substances, and chemical compounds. However, the complex mechanism associated with wound healing as a result of treatment has yet to be investigated [10]. Most in vivo studies have examined the effects of probiotics and keratinocytes on wound healing and the wound healing process. However, in the case of animal experiments, wound healing studies have focused on topical probiotic treatments to investigate wound closure, the prevention of pathogen infections, and the acceleration of wounded tissue repair [11,12,13]. In addition, these beneficial bacteria demonstrated antioxidant activity, an anti-inflammatory effect on the skin’s wound healing process, improved regulation of the skin immune system, and maintenance of cutaneous homeostasis [14,15].

Postbiotics, also known as ‘non-viable probiotics’ or ‘inactivated probiotics,’ are functional bioactive compounds that are generated by the fermentation of live bacteria or released after bacterial lysis [16]. Postbiotics produce useful effects, principally via immunomodulatory effects, protection against pathogens, and maintenance of intestinal homeostasis [17].

Although it has been well reported that applying probiotics on skin lesions accelerates wound healing, the effects of postbiotics using lactic acid bacteria (LAB) and the bioactive substances of metabolites associated with wound healing remain unclear. This study aimed to investigate the effectiveness of wound healing via the topical application of a heat-killed postbiotic, *Lactococcus chungangensis* CAU 1447, and to analyze potential metabolites, skin microbiota, various growth factors, chemokines, and cytokines involved in wound healing in a diabetic mouse model.

## 2. Materials and Methods

### 2.1. Preparation of the Postbiotic

Tryptic soy broth (TSB; Difco, BD, Sparks, MD) was used to culture *Lactococcus chungangensis* CAU 1447 and incubated at 30 °C for 48 h. After incubation, *Lc. chungangensis* CAU 1447 was yielded from TSB culture via centrifugation and washed with phosphate-buffered saline (PBS) (Lonza, Walkersville, MD, USA). Freeze-drying was conducted to produce dry *Lc. chungangensis* CAU 1447 cells. The freeze-dried CAU 1447 was reconstituted in PBS, diluted 10-fold, and then seeded on TSA (Tryptic soy agar) plate and incubated for 48 h to check the Colony Forming Unit (CFU). Freeze-dried CAU 1447 was adjusted to a concentration of 2 × 10^11^ CFU. Then, 1 mL of PBS was added and autoclaved at 100 °C for 30 min. After heat killing was completed, 1 mL of Eucerin (Beiersdorf, Mexico) was added and mixed to make a fresh ointment, where the final concentration was 1 × 10^11^ CFU/mL. The 100 µL of heat-killed *Lc. chungangensis* CAU 1447 combined with the Eucerin ointment was applied to each mouse for a once-daily treatment. The final concentration of the ointment was 1 × 10^10^ CFU/100 µL/wound area.

### 2.2. Bioactive Metabolite Analysis

The metabolite analysis of CAU 1447 was performed using High Performance Liquid Chromatography (HPLC). The CAU 1447 cells were reconstituted using PBS. Then, the composition of metabolites, such as organic, amino, and fatty acids, was determined via HPLC using an Ultimate 3000 (Thermo Scientific Dionex, Sunnyvale, CA, USA), which was provided by the College of Agriculture and Life Sciences, Seoul National University. The concentrations of these analyzed constituents were measured in milligrams per liter.

### 2.3. Animals

Six-week-old male C57BL/6J mice (*n* = 100) were obtained from the Central Lab Animal Incorporation (Korea) and acclimated for 7 days. Each group contained 25 mice divided into five cages. Each group of five mice was housed in a cage with free access to a nutritionally complete diet and water under a 12 h day and night cycle. The moisture and temperature were maintained at 55 ± 10% and 24 ± 2 °C, respectively. Animal studies were performed in line with the manual of the Korean Food and Drug Administration [18]. The ethical instructions in the manual were permitted by the Chung-Ang University Institutional Animal Care and Use Committee of the Laboratory Animal Research Center (2018-00022).

### 2.4. Streptozotocin-Induced Diabetes Model and Experimental Design

Diabetes was induced by multiple and repeated doses of streptozotocin via intraperitoneal (IP) injections (Sigma-Aldrich, St Louis, MO, USA) in a citrate buffer (pH 4.5). Streptozotocin (40 mg/kg/d) and a citrate buffer were administered to the mice on 5 consecutive days. To prevent fatal hyperglycemia, 10 % sucrose water was provided during the injection period. After 5 days of streptozotocin treatment, a glucometer was used to measure blood glucose levels, and only diabetic mice (those with a blood glucose level ≥ 350 mg/dL) were considered for this experiment. Moreover, in order to increase the accuracy of the reliability, exclusion criteria were established through the observation of the mouse cage environment and individual behavior. The diabetic mice were divided into four groups (n = 25/group) as follows: (1) a negative control group of diabetic mice, (2) a positive control group of diabetic mice treated with 100 µL PBS/wound area/day, (3) a Eucerin group of diabetic mice treated with 100 µL Eucerin/wound area/day, and (4) a CAU 1447 group of diabetic mice treated with a 100 µL mixture of Eucerin and CAU 1447/wound area/day. The predefined exclusion criteria were assessed during wound healing: (1) wound infection, (2) abnormal behavior, (3) spontaneous death, and (4) tissue destruction during dressing. In the CAU 1447 group, one mouse assigned on day 5 was excluded under the influence of environmental changes (abnormal behavior) in the same cage. The positive control group was treated with a volume of PBS equal to the amount that the other groups were treated with. All treatments continued for 10 days. For each treatment point after wound dressing, the mice were fasted for approximately 12 h before being anesthetized and sacrificed using CO_2_ inhalation. Their organs were dissected for further analysis.

### 2.5. Excisional Wound Procedures

The excisional wounds were created on the back of each mouse. Male, eight-week-old C57BL/6J mice were anesthetized with an intramuscular injection using xylazine (5 mg/kg/mouse) and ketamine (50 mg/kg/mouse). The back of each mouse was treated with hair removal cream, sterilized, and cleaned with 70% ethanol (Sigma, St. Louis, MO, USA). Four full-thickness excisional wounds were made on opposite sides of the upper and lower paravertebral muscles using a 6 mm biopsy punch instrument (Kai Medical, Seki, Japan). Diabetic wounds and surrounding skin biopsy tissues were obtained from each time point at 1, 3, 5, 7, and 10 days post wounding.

### 2.6. Measuring the Wound Closure

A digital caliper was used to measure the changes in wound size at each time point. The size and area of the wounds were calculated by measuring the horizontal and vertical lengths and comparing them to wound size on day 0. At each time point (days 1, 3, 5, 7, and 10), the wound proportion was analyzed and compared with the initial injury size.

### 2.7. Histological Analysis

Freshly wounded skin tissues were collected during each sacrifice. Wound tissue was fixed in 10% formalin, processed using the standard protocol, and cut into 4–5 µm sections. The sections were paraffin-embedded and stained with H&E to compare the histological change over time. Each tissue sample slide was observed under a light microscope and the skin tissue changes were assessed. The slides were evaluated at a minimum of three other located sections.

### 2.8. Cytokines, Growth Factors, and Chemokines Expression Levels from Wounded Skin Tissue

The mRNA expression levels of cytokines were analyzed using fresh and frozen wound tissue. Real-time PCR analysis was used to quantify inflammatory cytokines (TNF-α, IL-4, IL-6, and IL-10), growth factors (TGF-β1, VEGF, FGF, and PDGF), and chemokines (CCL-2/MCP-1 and CXCL4/PF4) related to wound healing. The wound tissue sample’s weight was set to about 25–30 mg and homogenized. The commercial RNeasy Mini Kit (Qiagen, Hilden, Germany) was used to isolate the total RNA from the wounded skin tissue per the manufacturer’s methodology. Extracted total RNA was added to 50 µL of RNase-free water and stored at −80 °C until analysis. A NanoQuant Spectrophotometer (Infinite 200: Tecan, Männedorf, Switzerland) was used to measure the concentration of the extracted total RNA. First, 1 µg of total RNA was reverse transcribed into cDNA using a PrimeScript™ 1st Strand cDNA Synthesis Kit (Takara Bio Inc, Shiga, Japan). Then, the real-time quantitative PCR 7500 Fast Real-Time PCR System and an SYBR Green PCR kit (Qiagen, Hilden, Germany) were used to quantify the mRNA expression levels. Fold changes were calculated using the 2^−^^△△Ct^ method. The primer sequences of inflammatory cytokine factors related to wound healing are expressed in Table 1. Control gene 36B4 was used.

### 2.9. Analysis of Myeloperoxidase Activity Assay

The peroxidase enzymes with MPO activity were estimated using an MPO assay kit (Colorimetric) (Abcam, Cambridge, UK) per the manufacturer’s guidelines. Briefly, fresh wounded skin tissues were collected at each time point and measured (the weight was 10 mg per mouse). Wounded skin tissues were kept in liquid nitrogen until their analysis. They were then homogenized, resuspended, and centrifuged. Then, aliquots of supernatant were transferred to sterilized micro-centrifuge tubes. MPO activity was indicated in micromoles. A microplate reader measured the absorbance at 412 nm.

### 2.10. DNA Extraction in Cutaneous Skin Wounds

Skin samples were collected from individual mice at each time point. Then, 1 mL of PBS was added and vortexed, after which, the supernatant was collected in a new 1.5 mL microtube. Centrifugation was performed to collect the bacterial pellet. Later, a FastDNA™ SPIN Kit (MP Biomedicals, LLC, Santa Ana, CA, USA) was used to extract bacterial DNA per the manufacturer’s guidelines.

### 2.11. 16S rRNA Gene Amplicon Sequencing

Paired-end read sequencing of the V3 and V4 variable regions of the 16S rRNA gene was amplified using PCR and performed on the Illumina Miseq platform (Illumina, San Diego, CA, USA). The raw amplicon data were truncated, denoised, dereplicated, stripped of the chimera, and merged with DADA2 [19] using QIIME2 (2020.2 release). Taxonomy classification was assigned at each phylogenetic level with the naïve Bayes classifier for primer sets. We used the Greengenes DB (13_8) trained database based on a 99% similarity. The raw data generated from metagenome sequencing were deposited in the National Center for Biotechnology Information Sequence Read Archive (NCBI; SRA) and are available under the accession numbers SRR15011482–SRR15011500 (BioProject PRJNA742783).

### 2.12. Statistical Analysis

To evaluate the statistical data, the wound size, mRNA expression levels, serum cytokines, and MPO activity were calculated, and flow cytometry analysis was performed using GraphPad Prism (v.7.0). Data are marked as means ± standard error of the mean (SEM). To compare the effects of more than two groups, the significant relative difference was analyzed with a two-way analysis of variance (ANOVA) using Bonferroni’s posthoc test. Any *p*-values < 0.05 were considered significant. The results from the amplicon sequences were exported into R and data were converted in the phyloseq package [20]. The sequence abundances were transformed into relative abundances by total reads and visualized using ggplot2 [21]. The reads for each sample were normalized using the total reads. The phyloseq package was used to analyze the alpha diversity metrics, including the observed, Chao1, and Shannon metrics. The PCoA of the beta diversity based on the Bray–Curtis distance was determined using the vegan package [22] and the permutational multivariate analysis of variance (PERMANOVA) was conducted with 999 permutations. Differential taxa abundances were measured in the phyloseq package [20]. A heatmap based on OTU taxa was determined using the phyloseq package and visualized using ggplot2. Other R packages used included plyr, readxl, ggpubr, and tidyverse.

## 3. Results

### 3.1. Bioactive Postbiotic Metabolites for Wound Healing

Potential metabolites were analyzed using high-performance liquid chromatography (HPLC). The concentrations of lactic acid (1138.34 mg/L), lysine (397.66 mg/L), acetic acid (284.71 mg/L), leucine (276.01 mg/L), and glycine (178.60 mg/L) were higher than those of other organic and amino acids. Likewise, for fatty acids, 1.22 mg/L of palmitic acid (hexadecanoic acid), 1.04 mg/L of stearic acid (octadecanoic acid), 0.76 mg/L of alpha-linolenic acid (omega-3), 0.44 mg/L of oleic acid (omega-9), 0.31 mg/L of palmitoleic acid (omega-7), 0.31 mg/L of myristic acid (tetradecanoic acid), and 0.10 mg/L of linoleic acid (omega-6) were quantified using HPLC analysis. Table 2 shows the quantifications of the various metabolites.

### 3.2. Postbiotics for Rapid Wound Closure

**Figure 1:**
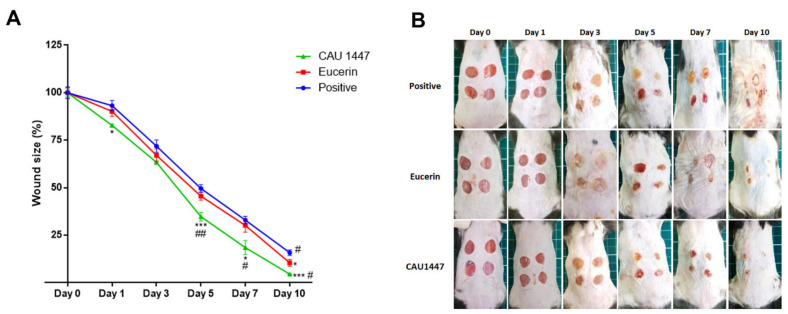
Effects of postbiotic administration on rapid skin wound closure. (**A**) Representative graph of wound size during the time course analysis. Wound area values were calculated using width and height measurements. (**B**) Representative images of the shaved back skin of C57BL/6 mice during the time course analysis after injury exposure. All values are given as mean ± standard error of the mean. The *p* -values are shown as *, *p* < 0.05; **, *p* < 0.005; ***, *p* < 0.0005. The statistical significance of differences is indicated as * vs Positive, and # Eucerin.

Full-thickness excisional circular wounds were created using a 6 mm biopsy punch of five mice from each group. To evaluate the changes in excisional wound size, a digital caliper was used to calculate the wound size at 1, 3, 5, 7, and 10 days after each injury. The proportion of wound closure was measured at each time point by calculating the unclosed wound area and representing it as a proportion of the initial wound area. The percentage of wound closure in all groups was calculated for days 1, 3, 5, 7, and 10 post wounding. As shown in Figure 1A, after day 3, there was a significantly sharper decline in mean wound size in the CAU 1447 group compared with the other groups (Figure 1A). From days 5 to 7, respectively, the wound size reduction rates were higher in the CAU 1447 group than the positive group (day 5, *p* = 0.0007; day 7, *p* = 0.0134) and the Eucerin group (day 5, *p* = 0.0073; day 7, *p* = 0.0414). Until day 10, the CAU 1447 group had the smallest mean wound size relative to those of the positive (*p* = 0.0001) and Eucerin (*p* = 0.0164) groups. This is reflected in the representative wound-size images taken after day 3 and shown in Figure 1B.

### 3.3. Effects of Postbiotic Administration on Histopathological Changes

**Figure 2:**
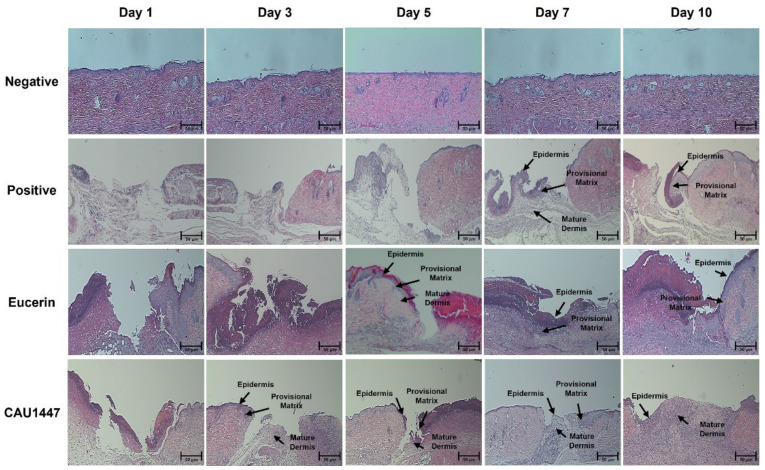
Effects of postbiotic administration on histopathological changes associated with wound healing. Hematoxylin and eosin staining of paraffin-embedded wounded skin sections at each time point. The arrows identify the epidermis, provisional matrix, and mature dermis. Bar: 50 µm.

The histopathological changes among all groups during the experimental period at each time point were examined using hematoxylin and eosin (H&E) staining. The appearance of the excisional wound was almost the same on day 1. Inflammation began in the dermis and there was moderate hemorrhaging and fibroblast proliferation in the positive, Eucerin, and CAU 1447 groups. On day 1, each group exhibited a wound surface layer that was lined by a narrow layer of collagen and a damaged keratinocyte layer with fibrin deposits. Additionally, neutrophils and scattered mononuclear cells began to appear in the wounded skin. Therefore, during this period, the differences in the wound-healing process were difficult to detect between the positive, Eucerin, and CAU 1447 groups. However, the extent of wound healing was the highest in the CAU 1447 group after day 3. Furthermore, fibro-proliferative tissue was first seen filling the wound in the CAU 1447 group. On days 5 and 7, faster re-epithelization and remodeling were observed in the CAU 1447 group than in the positive and Eucerin groups. Furthermore, epidermal regeneration and tissue granulation increased from day 5 to day 7. On day 10, the surface of the wound area was even and it had refilled with a new epithelium and well-formed granulation tissue. This was faster and more effective than in the Eucerin group (Figure 2).

### 3.4. Effects of Postbiotic Administration on mRNA Expression of Cytokines and Chemokines in Wounded Skin

**Figure 3:**
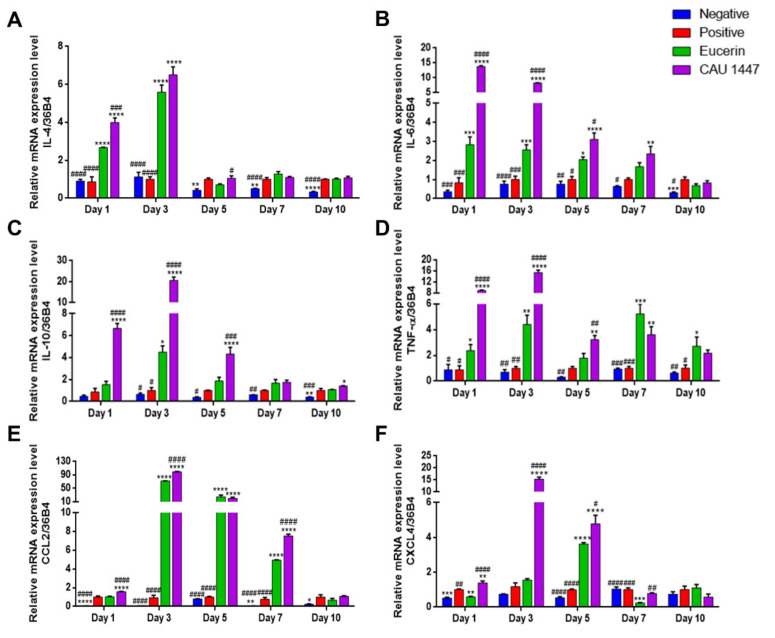
Effects of postbiotic administration on mRNA expression levels of cytokines and chemokines for wound healing. Differences in relative expression levels were determined with real-time PCR using wounded skin tissue. (**A**) IL-4, (**B**) IL-6, (**C**) IL-10, (**D**) TNF-α, (**E**) CCL2, and (**F**) CXCL4. Values are given as mean ± standard error of the mean. The *p*-values are shown as *, *p* < 0.05; **, *p* < 0.005; ***, *p* < 0.0005; ****, *p* < 0.0001. The statistical significance of differences is indicated as * vs Positive, and # Eucerin.

Differences in the relative mRNA expression levels of various cytokines and chemokines were observed for wound healing acceleration. Regarding cytokines, CAU 1447 application was associated with significantly higher IL-4 mRNA levels on days 1 and 3 when compared with those observed in the positive (day 1, *p* < 0.0001; day 3, *p* < 0.0001) and Eucerin (day 1, *p* = 0.0007) groups. On day 5, the CAU 1447 group had a significantly higher level of IL-4 mRNA than the Eucerin group (*p* = 0.0438). After that, the relative expression level of the CAU 1447 group was higher than that of the positive group, but this difference was not statistically significant (Figure 3A). In terms of IL-6 mRNA, the CAU 1447 group had the highest mean level relative to those of the positive (day 1, *p* < 0.0001; day 3, *p* < 0.0001; day 5, *p* < 0.0001) and Eucerin (day 1, *p* < 0.0001; day 3, *p* < 0.0001; day 5, *p* = 0.0019; day 7, *p* = 0.0036) groups until day 7. On day 10, the CAU 1447 and positive groups had similar mean IL-6 levels due to the late expression in the positive group during wound healing (Figure 3B). Furthermore, the mean IL-10 mRNA expression was highest in the CAU 1447 group until day 5 relative to the positive (day 1, *p* < 0.0001; day 3, *p* < 0.0001; day 5, *p* < 0.0001) and Eucerin (day 1, *p* < 0.0001; day 3, *p* < 0.0001; day 5, *p* = 0.0008) groups. After that, the mean IL-10 mRNA levels were continuously higher in the CAU 1447 group than the positive group on days 7 and 10 (*p* = 0.0211) (Figure 3C). Mean TNF-α mRNA expression levels were significantly higher in the CAU 1447 group than the positive group until day 7 (day 1, *p* < 0.0001; day 3, *p* < 0.0001; day 5, *p* = 0.0045; day 7, *p* = 0.0087) and the Eucerin group until day 5 (day 1, *p* < 0.0001; day 3, *p* < 0.0001; day 5, *p* = 0.0145) (Figure 3D).

Furthermore, in the CAU 1447 group, the mean level of CCL2 expression was significantly higher than that of the positive (*p* < 0.0001) and Eucerin (*p* < 0.0001) groups on day 1. After that, until day 7, the mean CCL2 level of the CAU 1447 group dramatically increased relative to those of the positive (day 3, *p* < 0.0001; day 5, *p* < 0.0001; day 7, *p* < 0.0001) and Eucerin (day 3, *p* < 0.0001; day 7, *p* < 0.0001) groups. On day 10, the mean CCL2 level in the CAU 1447 group remained the highest, but there were no significant differences between the groups (Figure 3E). In terms of the mean CXCL4 expression, until day 5, the CAU 1447 group had significantly higher levels than the positive (day 1, *p* = 0.0035; day 3, *p* < 0.0001; day 5, *p* < 0.0001) and Eucerin (day 1, *p* < 0.0001; day 3, *p* < 0.0001; day 5, *p* = 0.0220) groups. After day 7, the CAU 1447 group had similar or higher mean levels than the Eucerin group (*p* = 0.0019) (Figure 3F).

### 3.5. Effects of Postbiotic Administration on mRNA Expression of Growth Factors in Wounded Skin

**Figure 4:**
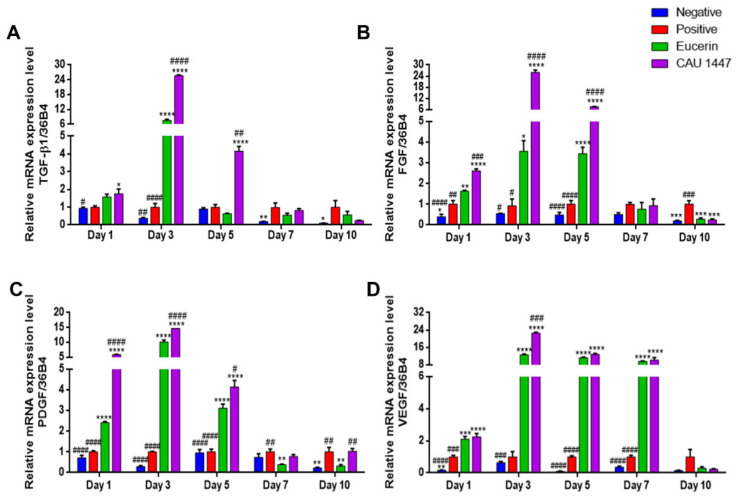
Effects of postbiotic administration on mRNA expression levels of growth factors for wound healing. Differences in relative levels were determined with real-time PCR using wounded skin tissue. (**A**) TGF-β1, (**B**) FGF, (**C**) PDGF, and (**D**) VEGF. Values are given as mean ± standard error of the mean. The *p* -values are shown as *, *p* < 0.05; **, *p* < 0.005; ***, *p* < 0.0005; ****, *p* < 0.0001. The statistical significance of differences is indicated as * vs positive, and # Eucerin.

We examined differences in relative growth factor mRNA expression levels that were associated with wound healing acceleration. The mean TGF-β1 mRNA expression level of the CAU 1447 group was significantly higher than that of the positive group on day 1 (*p* = 0.0211). Thereafter, the mean TGF-β1 level of the CAU 1447 group significantly increased relative to those of the positive (day 3, *p* < 0.0001; day 5, *p* = 0.0018) and Eucerin (day 3, *p* < 0.0001; day 5, *p* = 0.0017) groups until day 5 (Figure 4A). In terms of the mean FGF mRNA levels, until day 5, the CAU 1447 group had the same level as the positive (day 1, *p* < 0.0001; day 3, *p* < 0.0001; day 5, *p* < 0.0001) and Eucerin (day 1, *p* = 0.0003; day 3, *p* < 0.0001; day 5, *p* < 0.0001) groups (Figure 4B). Furthermore, until day 5, the CAU 1447 group had the highest mean PDGF level relative to those of the positive (day 1, *p* < 0.0001; day 3, *p* < 0.0001; day 5, *p* < 0.0001) and Eucerin (day 1, *p* < 0.0001; day 3, *p* < 0.0001; day 5, *p* = 0.0167) groups (Figure 4C). In terms of the mean VEGF mRNA expression, from day 1 to day 7, the CAU 1447 group had significantly higher levels than the positive (day 1, *p* < 0.0001; day 3, *p* < 0.0001; day 5, *p* < 0.0001; day 7, *p* < 0.0001) and Eucerin (day 3, *p* = 0.0004) groups (Figure 4D). Due to the late expression in the positive group, the mean relative mRNA level in the CAU 1447 group was similar or lower than that of the positive group on day 7 and day 10.

### 3.6. Postbiotic Alleviated Myeloperoxidase Activity

**Figure 5:**
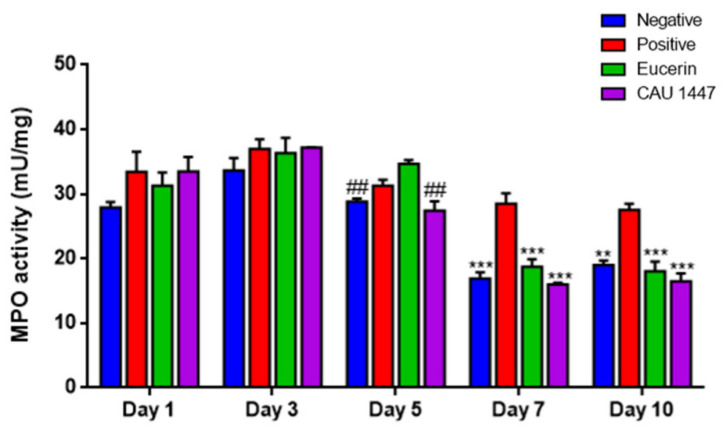
Effects of postbiotic administration on myeloperoxidase activity for wound healing. Myeloperoxidase concentrations were confirmed during the experimental period. Fresh frozen tissue (10 mg) was used and homogenated for the assay. Myeloperoxidase secretion was regulated via a topical postbiotic application on diabetic mice. Values are given as mean ± standard error of the mean with *p*-values. The *p*-values are shown as **, *p* < 0.005; ***, *p* < 0.0005. The statistical significance of differences is indicated as * vs positive, and # Eucerin.

The myeloperoxidase (MPO) analysis was conducted to confirm the changes in enzyme activity after the application of Eucerin and CAU 1447 ointments. On day 5, the mean MPO activity level of the CAU 1447 group was significantly lower than that of the Eucerin group (*p* = 0.0016). After that, until day 10, the mean MPO activity level of the CAU 1447 group declined significantly relative to that of the positive group (day 7, *p* = 0.0002; day 10, *p* = 0.0003). Additionally, the CAU 1447 group had a lower mean MPO activity level than the Eucerin group, but this difference was not statistically significant (Figure 5).

### 3.7. Microbial Diversity Displayed a Difference on Day 5 through Profiling of the Skin Microbiome

**Figure 6:**
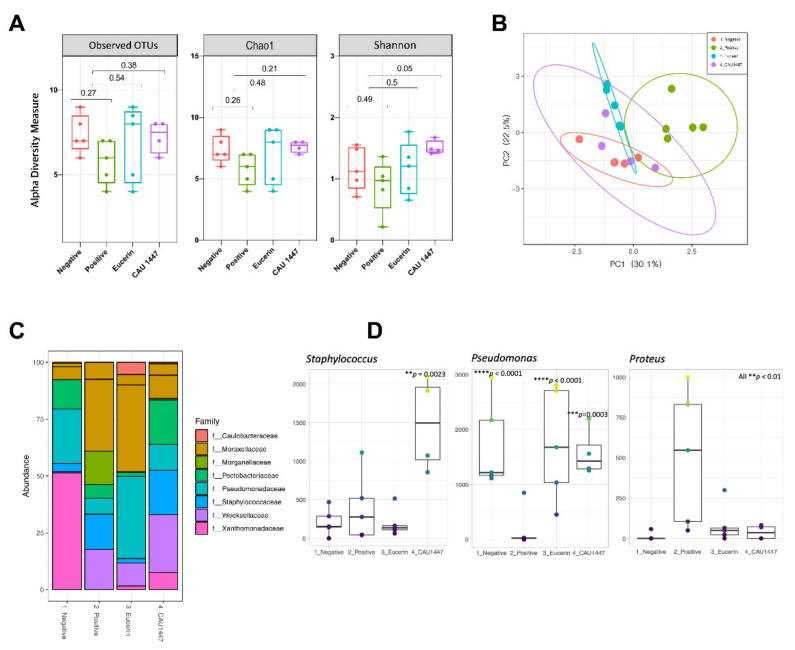
Effects of postbiotic administration on the abundance of skin microbiota. (**A**) Alpha diversity was calculated using the observed, Chao1, and Shannon indexes. (**B**) Principal component analysis plot generated using variance decomposition to reflect the clustering of skin microbial communities in all groups. (**C**) Taxonomic classification of the skin microbiome indicating the proportion of bacteria in each group at the family and genus levels. (**D**) Boxplots reflecting the abundances of *Staphylococcus*, *Pseudomonas*, and *Proteus* spp. in each group. The statistical significance of differences is indicated as * vs Positive.

A microbiome analysis to determine the effect of the application of CAU 1447 showed that the skin bacteria composition was significantly different on day 5. The alpha diversity value of the observed OTUs and Chao 1 and Shannon diversity indexes showed that the observed OTUs and Shannon indexes of the CAU 1447 group was high in comparison to the positive group (observed OTUs index: *p* = 0.38 between positive and CAU 1447; Chao 1 index: *p* = 0.21 between positive and CAU 1447; Shannon index: *p* = 0.05 between positive and CAU 1447) (Figure 6A). Through a principal coordinates analysis (PCoA) plot using Bray–Curtis distances, we observed that the positive group formed different clusters from the negative, Eucerin, and CAU 1447 groups (PERMANOVA: Bray–Curtis distance *p* < 0.05) (Figure 6B). The CAU 1447 group revealed bacterial composition patterns that were similar to those of the negative group. The mean relative abundances of the skin bacterial composition of some families and genera were different between all groups on day 5. Family-level taxonomic classification of the skin microbiome revealed a significant difference in the taxon composition between the positive and CAU 1447 groups, including microbiota associated with taxa such as *Pseudomonadaceae* and *Staphylococcaceae*. The relative abundances of *Staphylococcus* spp. and *Pseudomonas* spp. were higher in the CAU 1447 group than in the positive group. However, the relative abundance of *Proteus* spp. was higher in the positive group (Figure 6C). Figure 6D presents the boxplots reflecting the abundances of *Pseudomonas*, *Staphylococcus*, and *Proteus* spp. in each group.

## 4. Discussion

Many studies were recently conducted on the potential helpful effects of probiotics on wound healing. In the current study, the application of a postbiotic accelerated wound healing activity on an excisional wound in a diabetic animal model with delayed angiogenesis and wound healing was investigated. These results are supported by a study of *Lactobacillus* strains indicating that they protect against the pathogen invasion in wounds and stimulate the release of many growth factors and cytokines in cutaneous wound healing [23].

Our research showed that several bioactive metabolites from the profiling analysis of the postbiotic were involved in wound healing. Through the analysis of the detected potential metabolites, we found that these substances were related to improved wound healing by regulating inflammation or skin tissue regeneration. Both palmitic acid and palmitoleic acid, which increase anti-inflammatory activity and inhibit pro-inflammatory cytokine production, are related to the wound healing mechanism [24]. In addition, a chronic disease related to TNF-α expression showed impaired wound healing and tissue breakdown [25,26]. In vivo, improved wound healing through anti-inflammatory activity was demonstrated using palmitoleic acid [27]. Likewise, other studies have demonstrated that stearic acid and linoleic acid (omega-3) play a role in vessel formation and tissue regeneration in wound healing [28,29]. Consequently, the potential bio-compounds from heat-killed CAU 1447 may seem to play an assistant role to help wound healing through the application of CAU 1447.

Generally, wound healing and angiogenesis occur naturally after a skin injury and wound size reduces over time. Here, after the lesions appeared and the wounded areas were treated with a postbiotic mixed with Eucerin, the ratio of wound size reduction was the largest on days 3 and 5. Most wounds were nearly restored by the last day. A study elucidated that *Lactobacillus plantarum* accelerated wound healing in skin lesions and the healing percentage in skin lesions was significantly increased between day 3 and day 7 [30]. Moreover, *Lactobacillus casei* demonstrated a positive effect on wound healing as it decreased the wound area and stimulated the wound healing process in a diabetic animal model [31]. The postbiotic showed a positive effect on wound healing in the skin tissue morphology. The extent of wound healing was highest with the postbiotic, and the wound area was first filled with fibro-proliferative tissue.

The inflammatory phase, the first stage of the wound healing process, involves the secretion of various growth factors and cytokines by neutrophils and macrophages. In this study, the group that received the postbiotic showed levels that were similar to that of the positive group until day 3. After day 3, the MPO levels significantly decreased compared to the positive and Eucerin groups. The overproduction of MPO activates ROS production, postponing wound healing activity [32]. Some studies have demonstrated that *Lactobacillus* strains decrease the MPO levels in wounded tissue. For instance, in treatment with heat-killed and live *Lb. rhamnosus*, the probiotic Lacidofil (composed of *Lb. helveticus* R0052, *Lb. rhamnosus* R0011, and *Lb. reuteri*) decreased the MPO level [33,34,35]. Therefore, a topical postbiotic application may accelerate wound healing by decreasing MPO activity from day 5 onward and rapidly proceed to the next stage, namely, the proliferation phase, and beyond the inflammatory phase.

Essential mediators of wound healing, such as chemokines, growth factors, and cytokines, were adjusted to determine the crucial physiological and molecular mechanisms in wound repair [36]. VEGF, PDGF, FGF, and TGF-β1 are representative growth factors that play important roles in each phase of the wound healing process, including angiogenesis, granulation of tissue formation, re-epithelialization, collagen deposition, and inflammation [37]. Moreover, the initial growth factors released by wound inflammatory cells at the wound site cause fibroblasts to secrete growth factors for wound healing [38]. Treatment with a postbiotic significantly increased the mRNA expression levels of VEGF, PDGF, FGF, and TGF-β1 from wounded skin tissue on the day after the wound occurred and on day 3. One study suggested that treatment with individual growth factors, such as PDGF and FGF, accelerated and improved the healing process in diabetic mice [39]. In addition, *Lb. rhamnosus* GG lysates improved the re-epithelialization in keratinocytes and increased the expression level of VEGF, which is an elemental initiator of angiogenesis, in a gastric ulcer model [40,41]. Another study suggested that the compounds of *Lb. reuteri* extracts increased TGF-β1 expression levels and accelerated the wound-healing process [42,43]. Therefore, a topical postbiotic application may accelerate wound healing by upregulating growth factor expression.

Various chemokines and cytokines are essential at each stage of the wound-healing process [44]. Among chemokines, the chemoattractant proteins CCL2 and CXCL4 regulate tissue formation, inflammation granulation, and re-epithelialization in excisional wounds via macrophage infiltration [37]. In this study, the application of the postbiotic increased the CCL2 and CXCL4 expression levels compared to the positive and Eucerin groups. In one study, CCL2 and CXCL4 treatment accelerated the wound healing process in a diabetes model [45]. Cytokines adjust the phase of the wound healing process, as well as the differentiation, migration, regulation of cell growth, and proliferation [46].

Notably, we observed a high mRNA expression and high concentrations of IL-4, IL-6, IL-10, and TNF-α on wounded skin tissue in the postbiotic-treated group compared to the other groups. This result is supported by several previous studies demonstrating that treatment with some probiotic strains, such as *Lb. salivarius* LA307, *Lb. rhamnosus* LA305, *Lb. sakei*, *Lb. plantarum*, and *Bifidobacterium bifidum* PI22, activated collagen deposition, tissue remodeling, and angiogenesis for rapid wound healing through cytokine secretion [47,48,49,50]. Therefore, the evidence shows that CAU 1447 regulates the expression of cytokines, chemokines, and growth factors, and therefore may promote wound healing through the regulation of wound healing factors.

Skin microbiota changes have significant implications for cutaneous wound healing [51]. Most publications describing wound-healing-related microbiota have focused on specific organism changes and microbial burden. Therefore, via microbiome studies, much remains to be uncovered about the functions of skin microbiome diversity in wound healing [52]. In our animal model, we observed the presence of *Pseudomonas* spp., *Staphylococcus* spp. and *Proteus* spp., all of which naturally exist in the human skin microbiome [51,53]. In our model of diabetic mice induced by streptozotocin, the bacterial profile was characterized by the individualized abundances on day 5. Other studies have demonstrated *Proteus* spp. to be present in high proportions in chronic wounds and diabetic foot ulcers, with decreases in *Proteus* abundance achieved via treatment with *Lb. reuteri* DSM [54,55,56]. In the present study, there was a low proportion of *Proteus* spp. in the CAU 1447 group. On the other hand, compared with the positive group, higher proportions of *Pseudomonas* spp. were associated with CAU 1447 application. High proportions of *Pseudomonas* spp. were associated with accelerated wound healing via enhanced epithelialization [52,57]. Additionally, high proportions of *Staphylococcus* spp. were associated with inhibited pathogen invasion and suppressed skin inflammation with regard to wound healing [51]. Treatment with *Lc. plantarum* GMNL6 has been associated with higher amounts of *Staphylococcus* spp. [58]. Therefore, *Lc. chungangensis* CAU 1447 application may promote wound healing and suppress pathogen invasion by accelerating increased proportions of *Pseudomonas* spp. and *Staphylococcus* spp. in the skin microbiome.

## 5. Conclusions

Wound dressings infused with postbiotics effectively facilitated healing in diabetic mice via the early expression of healing factors and cytokines (IL-4, IL-6, IL-10, and TNF-α), growth factors (TGF-β1, VEGF, PDGF, and FGF), and chemokines (CCL2 and CXCL4). Additionally, reductions in MPO activity and changes in the composition of the skin microbiome were associated with accelerated healing. These results suggest topical *Lc. chungangensis* CAU 1447 application as a promising strategy for facilitating wound healing, particularly in the context of diabetes mellitus.

## Figures and Tables

**Table 1:** Primers used for the real-time qPCR.

Gene	Sequence	Primer
IL-4	Sense (5′ to 3′)	GGCATTTTGAACGAGGTCAC
	Anti-sense (3′ to 5′)	AAATATGCGAAGCACCTTGG
IL-6	Sense (5′ to 3′)	CTACCCCAATTTCCAATGCT
	Anti-sense (3′ to 5′)	ACCACAGTGAGGAATGTCCA
IL-10	Sense (5′ to 3′)	TGAATTCCCTGGGTGAGAAG
	Anti-sense (3′ to 5′)	TGGCCTTGTAGACACCTTGG
TNF-α	Sense (5′ to 3′)	TCCCAGGTTCTCTTCAAGGGA
	Anti-sense (3′ to 5′)	GGTGAGGAGCACGTAGTCGG
TGF-β1	Sense (5′ to 3′)	GCTACCATGCCAACTTCTGT
	Anti-sense (3′ to 5′)	CGTAGTAGACGATGGGCAGT
VEGF	Sense (5′ to 3′)	CCACGTCAGAGAGCAACATCA
	Anti-sense (3′ to 5′)	TCATTCTCTCTATGTGCTGGCTTT
PDGF	Sense (5′ to 3′)	TCAAGCTCGGGTGACCATTC
	Anti-sense (3′ to 5′)	ACTTTCGGTGCTTGCCTTTG
FGF	Sense (5′ to 3′)	CAGCACAATGGCAGGCAAAT
	Anti-sense (3′ to 5′)	CATGGGGAGGAAGTGAGCAG
CCL2/MCP-1	Sense (5′ to 3′)	TTAAAAACCTGGATCGGAACCAA
	Anti-sense (3′ to 5′)	GCATTAGCTTCAGATTTACGGGT
CXCL4/PF4	Sense (5′ to 3′)	CGATGGAGATCTTAGCTGTGTG
	Anti-sense (3′ to 5′)	CATTCTTCAGGGTGGCTATGAG

**Table 2:** Metabolite concentrations of heat-killed *Lactococcus chungangensis* CAU 1447.

Organic/Amino Acid	Concentration (mg/L)	Fatty Acid	Concentration (mg/L)
Lactic acid	1138.34	Caproic acid (hexanoic acid)	0.094
Acetic acid	284.71	Lauric acid (dodecanoic acid)	0.087
Aspartic acid	77.19	Myristic acid (tetradecanoic acid)	0.307
Glutamic acid	272.43	Palmitic acid (hexadecanoic acid)	1.220
Asparagine	105.88	Palmitoleic acid (omega-7)	0.311
Serine	112.99	Stearic acid (octadecanoic acid)	1.038
Glutamine	30.72	Oleic acid ^†^ (omega-9)	0.435
Histidine	44.81	Linoleic acid (omega-6)	0.099
Glycine	178.60	Alpha-linolenic acid (omega-3)	0.764
Alanine	127.05		
GABA	46.81		
Valine	113.16		
Methionine	61.57		
Tryptophane	57.33		
Phenylalanine	92.26		
Isoleucine	61.15		
Leucine	276.01		
Lysine	397.66		
Proline	16.58		

## Data Availability

Through the corresponding author, the data represented in this study are accessible on requirement.
